# The characterization of amphibian nucleoplasmins yields new insight into their role in sperm chromatin remodeling

**DOI:** 10.1186/1471-2164-7-99

**Published:** 2006-04-28

**Authors:** Lindsay J Frehlick, José María Eirín-López, Erin D Jeffery, Donald F Hunt, Juan Ausió

**Affiliations:** 1 Department of Biochemistry and Microbiology, University of Victoria, Petch Building, Victoria, BC, V8W 3P6, Canada; 2 Departamento de Biología Celular y Molecular, Universidade da Coruña, Campus de A Zapateira s/n, E-15071, Spain; 3 Department of Chemistry and Pathology, University of Virginia, Charlottesville, VA 22901, USA

## Abstract

**Background:**

Nucleoplasmin is a nuclear chaperone protein that has been shown to participate in the remodeling of sperm chromatin immediately after fertilization by displacing highly specialized sperm nuclear basic proteins (SNBPs), such as protamine (P type) and protamine-like (PL type) proteins, from the sperm chromatin and by the transfer of histone H2A-H2B. The presence of SNBPs of the histone type (H type) in some organisms (very similar to the histones found in somatic tissues) raises uncertainty about the need for a nucleoplasmin-mediated removal process in such cases and poses a very interesting question regarding the appearance and further differentiation of the sperm chromatin remodeling function of nucleoplasmin and the implicit relationship with SNBP diversity The amphibians represent an unique opportunity to address this issue as they contain genera with SNBPs representative of each of the three main types: *Rana *(H type); *Xenopus *(PL type) and *Bufo *(P type).

**Results:**

In this work, the presence of nucleoplasmin in oocyte extracts from these three organisms has been assessed using Western Blotting. We have used mass spectrometry and cloning techniques to characterize the full-length cDNA sequences of *Rana catesbeiana *and *Bufo marinus *nucleoplasmin. Northern dot blot analysis shows that nucleoplasmin is mainly transcribed in the egg of the former species. Phylogenetic analysis of nucleoplasmin family members from various metazoans suggests that amphibian nucleoplasmins group closely with mammalian NPM2 proteins.

**Conclusion:**

We have shown that these organisms, in striking contrast to their SNBPs, all contain nucleoplasmins with very similar primary structures. This result has important implications as it suggests that nucleoplasmin's role in chromatin assembly during early zygote development could have been complemented by the acquisition of a new function of non-specifically removing SNBPs in sperm chromatin remodeling. This acquired function would have been strongly determined by the constraints imposed by the appearance and differentiation of SNBPs in the sperm.

## Background

Nucleoplasmin was initially isolated from African clawed frog (*Xenopus laevis*) egg extracts and it was described as an acidic chaperone protein capable of assembling nucleosomes [1,2]. Indeed, it represents the "archetypal" molecular chaperone [3] and is certainly the first of a long list of chromatin assembly complexes that have been, and still are, the focus of much research attention [4,5]. In the egg, nucleoplasmin is found natively bound to histones H2A and H2B, facilitating the storage of these proteins [3,6,7].

Nucleoplasmin is the most abundant protein in *Xenopus *oocytes. It is a highly thermostable protein and is very rich in acidic amino acids (approx pI = 5). It consists of approximately 200 amino acids and exists as a pentamer [2,8]. The first 120 N-terminal amino acids contain a small acidic cluster and exhibit a highly organized secondary and tertiary structure and constitute the "core" of the protein that is responsible for the formation of the pentameric structure of the molecule. By contrast, the last 80 C-terminal amino acids are sensitive to protease digestion, highly unstructured, and hence correspond to the "tails" of the protein. These regions contain two polyglutamic clusters, one of which spans 20 amino acids, and a bipartite nuclear localization signal [9].

*In vivo*, nucleoplasmin has been shown to mediate the *X. laevis *sperm decondensation in the egg cytoplasm that takes place immediately after fertilization [10]. This process involves the binding of the *X. laevis *SNBPs and transfer of H2A-H2B to the sperm chromatin [10]. The protein exchange is enhanced by the dramatic increase in phosphorylation undergone by the protein [11] (up to 14–20 phosphates per nucleoplasmin monomer [12]) during the maturation of the oocyte into the unfertilized egg [7]. High levels of phosphorylation remain until the mid-blastula transition (MBT), following which the levels drop off [11].

SNBPs can be classified in three major groups (H, P and PL) according to their compositional and structural organization [13,14]. The histone type H consists, as its name indicates, of proteins which are very similar to the histones that are found associated with DNA in somatic chromatin complexes [15]. The protamine type P includes a group of highly specialized arginine-rich sperm chromosomal proteins of relatively small molecular mass (≤ 10,000) [16]. The protamine-like type encompasses a highly structurally heterogeneous group of proteins with intermediate composition between protamines and histones (lysine- and arginine-rich) which are evolutionarily related to histone H1 [13,17].

The SNBPs of *X. laevis *consist of a structurally heterogeneous mixture of protamine-like proteins named SP1-SP6 that coexist with an H2A-H2B deficient complement of histones H3 and H4 [18]. It is important to mention though, that this composition varies significantly between different species of *Xenopus *which in some instances only contain SP1-2 (such as in *X. tropicalis*) and more often than not contain a stoichiometric complement of nucleosomal H2A-H2B and H3-H4 [18].

In contrast to the SP1-SP6 proteins from *X. laevis *that have an amino acid composition intermediate between histones and protamines and hence belong to the PL type [13], the toad *Bufo *contains protamines in its sperm that are very rich in arginine and can be considered representatives of the vertebrate P type SNBP [19]. In the frog *Rana*, the mature sperm contains histones that are almost indistinguishable from the somatic counterparts as is characteristic of the H type [20,21].

If nucleoplasmin is involved in sperm chromatin remodeling during fertilization [22], the existence of three main types of structurally and compositionally different SNBP raises the question of whether there are three different nucleoplasmins specific for each of these types or whether, in contrast to SNBPs, nucleoplasmin is a highly conserved molecule. Amphibians provide a unique opportunity to address this question as the group contains representative organisms of each of the main SNBP types.

In this paper we have characterized the structure of the *Xenopus *nucleoplasmin gene as well as the complete sequences of both the *Bufo marinus *and *Rana catesbeiana *nucleoplasmin cDNAs. The analysis of these sequences shows that nucleoplasmin is indeed a highly conserved protein that is closely related to mammalian NPM2, and suggests that its functional role goes beyond chromatin assembly immediately after fertilization, into the early stages of development. We propose that nucleoplasmin may have acquired the function of SNBP removal after fertilization in many vertebrates because of the appearance and vertical evolution of SNBPs.

## Results

### Ubiquitous presence of nucleoplasmin in the eggs of amphibians

**Figure 1:**
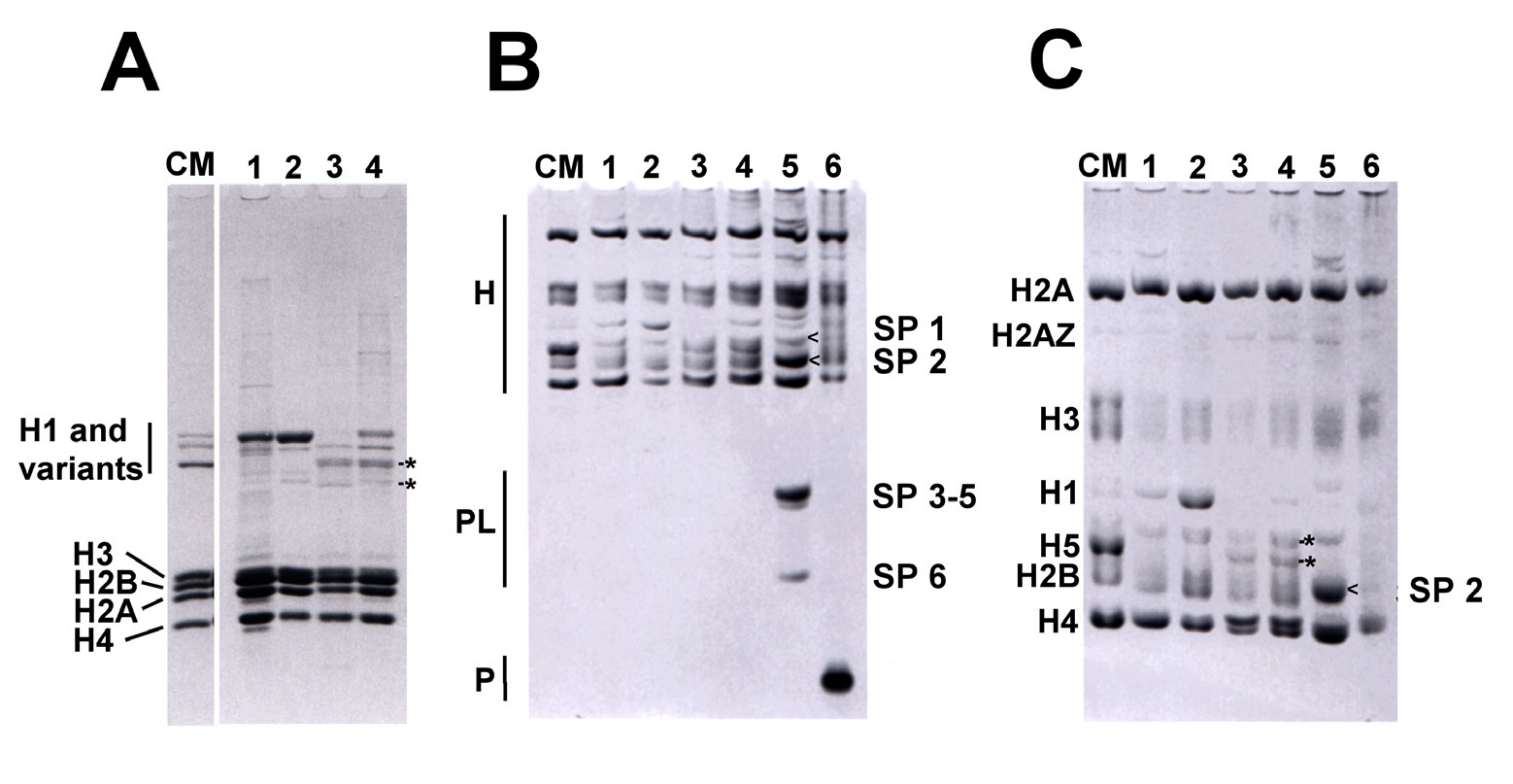
**Electrophoretic characterization of *R. catesbeiana*, *X. laevis *and *B. marinus *SNPBs**. Characterization of histones and SNBPs extracted from different tissues by: A) SDS-PAGE; B) AUT-PAGE run for a short time duration to separate histones (H), protamine-like proteins (PL) and protamines (P); and C) AUT-PAGE run for a longer duration to separate histones. Lane 1: *R. catesbeiana *liver; lane 2: *R. catesbeiana *blood; lane 3: *R. catesbeiana *sperm; lane 4: *R. catesbeiana *testes; lane 5: *X. laevis *testes; lane 6: *B. marinus *testes. CM; Chicken erythrocyte histones used as a histone marker. The SNBPs of *X. laevis *in lane 5, called sperm-specific proteins (SP1-6), are labeled and arrows (<) are used when needed to clearly indicate which bands the labels refer to. The asterisks point to the sperm-specific histone H1 complement in *R. catesbeiana*.

As it can be seen in Figure 1A–C, *R. catesbeiana *sperm contains only core histones that, except for the histone H1 counterpart, exhibited identical electrophoretic mobility to those of somatic histones (Fig. 1A–C, lane 3). Indeed, the sperm of this amphibian has been shown to consist of core histones that have amino acid compositions identical to those of somatic tissues and a set of highly specific linker histones with similar composition to histone H1 [21].

In contrast to *R. catesbeiana*, the SNBPs of *X. laevis *in testis showed the presence of a complex mixture of SNBPs of the PL type (SP1-SP6) [18] (Fig. 1B, lane 5), which in the mature sperm coexist with a H3-H4 complement. Alternatively, *B. marinus *contained a typical vertebrate protamine [19,20] (Fig. 1B, lane 6). Thus, the protein composition of the sperm chromatin of these organisms is very different.

With the exception of *X. laevis *nucleoplasmin, which has been extensively characterized and has been for many years used as a generic prototype for nucleoplasmin, only fractional information is available on *Bufo *nucleoplasmin [23] and very little is known about its existence in *Rana *[24]. Therefore, we took advantage of an antibody developed in our lab against recombinant *X. laevis *nucleoplasmin to identify the nucleoplasmin-like proteins in egg extracts of these two organisms. The candidate bands from gels were then used to obtain some partial protein sequence information in order to further confirm the bands identity and to produce primers that would allow us to obtain the complete cDNA sequences of these proteins.

**Figure 2:**
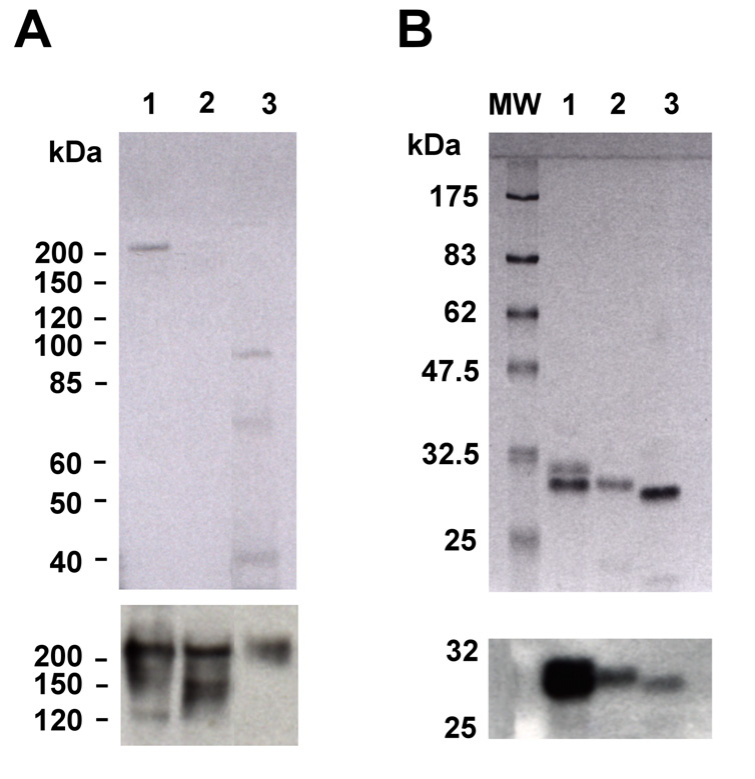
**Western blot analysis of egg extracts and purified nucleoplasmin proteins**. A) SDS-PAGE of heat soluble egg extracts from: Lane1, *X.laevis*; lane 2, *B. marinus*; lane 3, *R. catesbeiana*. Egg extract aliquots were mixed with an equal volume of 2 × SDS sample buffer and loaded in the gel without any previous boiling. Under these conditions the nucleoplasmin protein retains its pentameric conformatiom [25]. Molecular weights are a PageRuler Protein Ladder (Fermentas Life Sciences, Burlington, ON) B) SDS-PAGE of nucleoplasmin proteins purified from the extracts of: Lane1, *X. laevis*; lane 2, *B. marinus*; lane 3, *R. catesbeiana*. Samples were boiled in SDS (0.1%) sample buffer for 10 minutes before loading on the gel to separated nucleoplasmin proteins into their monomeric forms. MW is a prestained broad range molecular weight protein marker (New England Biolabs, Ipswich, MA). Western blot analysis was done using a polyclonal antibody elicited against recombinant *X. laevis *nucleoplasmin [28] and the results are shown in the lower panels of both A) and B) below the corresponding gels.

One of the distinctive features of nucleoplasmin is its ability to retain its native pentameric conformation (Mr = 110,000) in the presence of the SDS used in the sample buffer of SDS-PAGE [25]. The monomeric subunit can only be visualized after extensive (*ca*. 10 min) boiling in the presence of the SDS (1%) sample buffer. We exploited this highly characteristic property in order to analyze the egg extracts from *X. laevis*, *B. marinus *and *R. catesbeiana*. Figure 2 shows the results of unboiled (Fig. 2A) and boiled (Fig. 2B) extracts separated by SDS-PAGE (upper panels). The presence of nucleoplasmin was assessed by Western blot analysis using an antibody against *X. laevis *recombinant nucleoplasmin (Fig. 2, lower panels) [26]. For all three extracts a higher molecular weight form corresponding to the pentamer was seen in the unboiled samples (Fig. 2A) which dissociated into monomers following boiling in SDS sample buffer (Fig. 2B). Note that *X. laevis *and *B. marinus *exhibited a smeared band in the region of the SDS-PAGE corresponding to the pentameric form, a fact that can be attributed to either differences in the extent of phosphorylation or the incorrect folding of the pentamer. In addition, due to its highly charged nature (polyglutamic acid tracts and phosphate residues), nucleoplasmin is known to have aberrant migration on SDS-PAGE gels.

Although the presence of nucleoplasmin in *Bufo *had already been well documented [23], the presence of a nucleoplasmin-like equivalent protein in *Rana *had never been demonstrated, despite the fact it was known that *Rana *egg extracts had the ability to decondense demembranated sperm nuclei from *Xenopus *[24]. The results of Figure 2 encouraged us to pursue the isolation of such a protein and also to determine the hitherto unknown primary structure of *Bufo *nucleoplasmin. To this end, nuclear extracts were purified as described in the experimental section and the extent of purity of the fractions was monitored by SDS-PAGE Western analysis. Bands for the *B. marinus *and *R. catesbeiana *nucleoplasmins, such as those shown in Figure 2, were excised and analyzed by mass spectroscopy to obtain partial protein sequence information. This information, together with the nucleotide sequence available from the genome draft of *X. tropicalis *for the nucleoplasmin gene (assigned name: ESTEXT_FGENESH1_PG.C_340042 at [27]), was used to generate primers to be used in conjunction with mRNA prepared from both *R. catesbeiana *and *B. marinus *egg extracts.

### The amphibian nucleoplasmin gene contains multiple exons and encodes highly conserved proteins

**Figure 3:**
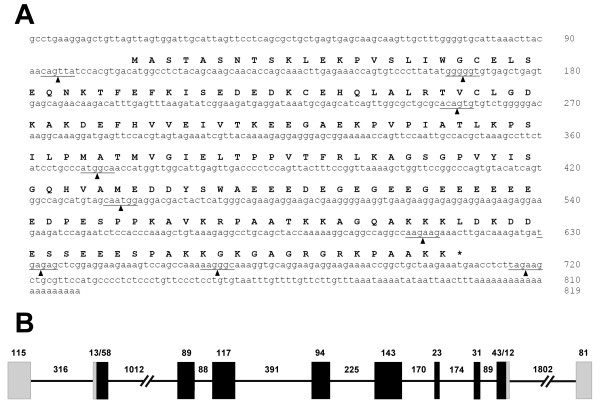
***X. tropicalis *nucleoplasmin gene**. A) Nucleotide sequence and corresponding protein sequence of a nucleoplasmin cDNA from *X. tropicalis *[GenBank: NM_001016938]. The arrow heads and lines indicate the sites of insertion of the different introns. B) Schematic representation of the organization and structure of the gene. Exons are schematically represented by solid boxes, with black representing translated regions and grey representing untranslated regions. Introns are indicated by black lines. The numbers indicate the basepair size of the regions below them.

As it has been mentioned in the previous section, the current availability of the *X. tropicalis *genome draft has proven to be very useful in achieving some of the objectives pursued in this work. Figure 3A shows the coding region corresponding to the nucleoplasmin gene of *X. tropicalis *[GenBank: NM_001016938] and the corresponding organization of the gene, which contains of 9 introns, is shown in Figure 3B. A very similar organization was found in the case of the *X. laevis *nucleoplasmin gene when it was analyzed using the genomic DNA of this species and primers designed based on the cDNA sequence (results not shown).

**Figure 4:**
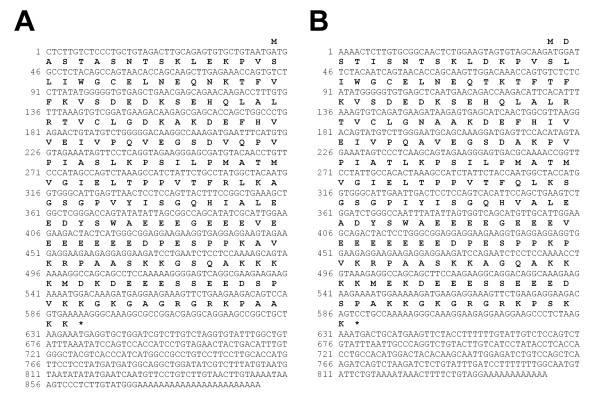
**Coding nucleotide sequences of *B. marinus *and *R. catesbeiana *nucleoplasmin**. The nucleotide sequences determined in this study and translated protein sequences of nucleoplasmin cDNAs from A) *B. marinus *[GenBank: DQ340657] and B) *R. catesbeiana *[GenBank:DQ340656] are shown.

We also established the complete cDNA sequences for one of the nucleoplasmin genes from each *B. marinus *[Genbank: DQ340657] and *R. catesbeiana *[Genbank: DQ340656] which were submitted to GeneBank December, 2005 (Fig. 4A–B). The precise number of nucleoplasmin genes in each genome has not yet been determined. However, two different cDNAs have been isolated from *X. laevis *that encode for two almost identical forms of the protein, except for the fact that one of them is 9 amino acids shorter [28] than the other [29]. It is thus possible that at least one other cDNA sequence besides those shown in Figure 4 is present in each of these species. Nevertheless, mass spectroscopic determination of the nucleoplasmin proteins purified by us from the egg extracts, confirmed that the proteins encoded by the cDNAs shown in Figure 4 correspond to the main translated mRNA forms (results not shown).

**Figure 5:**
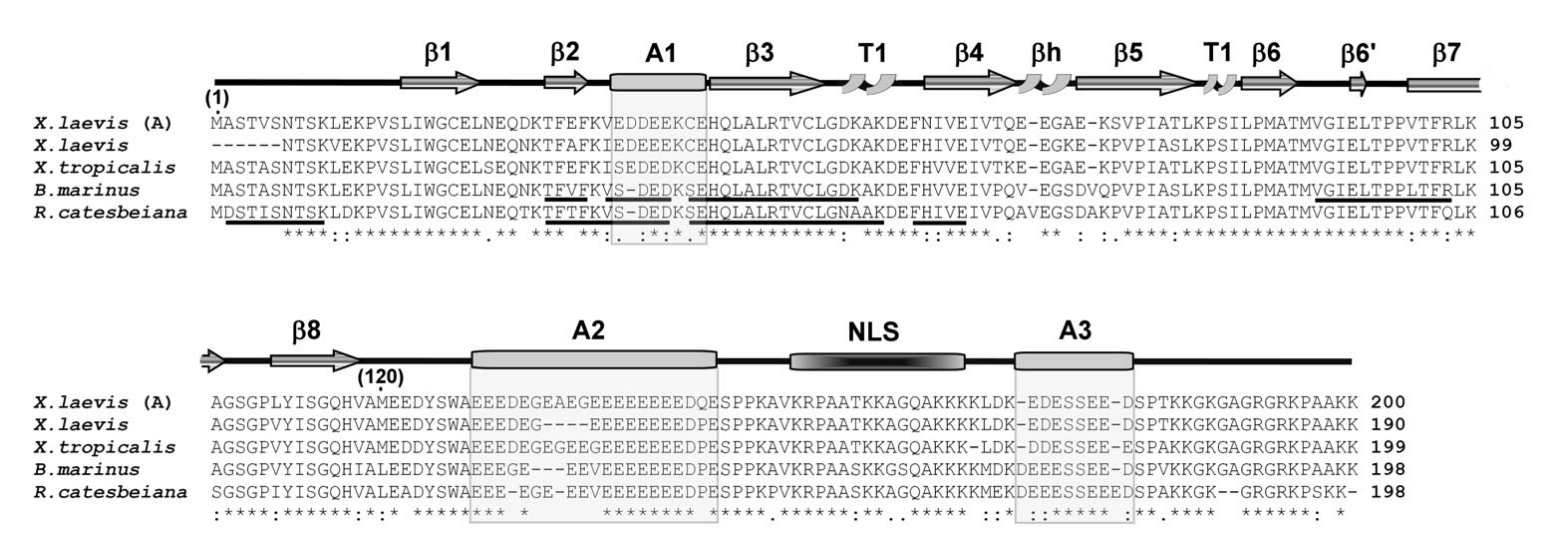
**Protein sequence alignment of amphibian nucleoplasmins**. The primary structures of nucleoplasmin from *X. laevis *(A) [60] [GenBank: X04766], *X. laevis *(Burglin et al., 1987) [GenBank: CAA68363], *X. tropicalis *[GenBank: NP_001016938], *B. marinus *and *R. catesbeiana *are shown. Identical amino acids are denoted by an asterisk, highly similar residues by a colon, and less similar residues by a period, as determined by CLUSTAL W software. The partial protein sequences of *B. marinus *and *R. catesbeiana *determined by mass spectroscopy peptide sequencing are underlined. The highly structured N-terminal protein core spans amino acids 1–120 and has β sheets (β1–8), two type 1 turns (T1) and a β hairpin (βh) [8]. The other boxes represent the A1, A2, A3 polyglutamic tracts and the bipartite nuclear localization signal (NLS), as indicated.

The comparison of the amino acid sequences for the different amphibian nucleoplasmin proteins is shown in Figure 5. As it can be seen, the protein sequence is extremely conserved (approximately 75 % identity) among the different amphibian species analyzed. Hence, it is to be expected that these proteins all have a common closely related function across these species.

### Amphibian nucleoplasmin is closely related to mammalian NPM2

**Figure 6:**
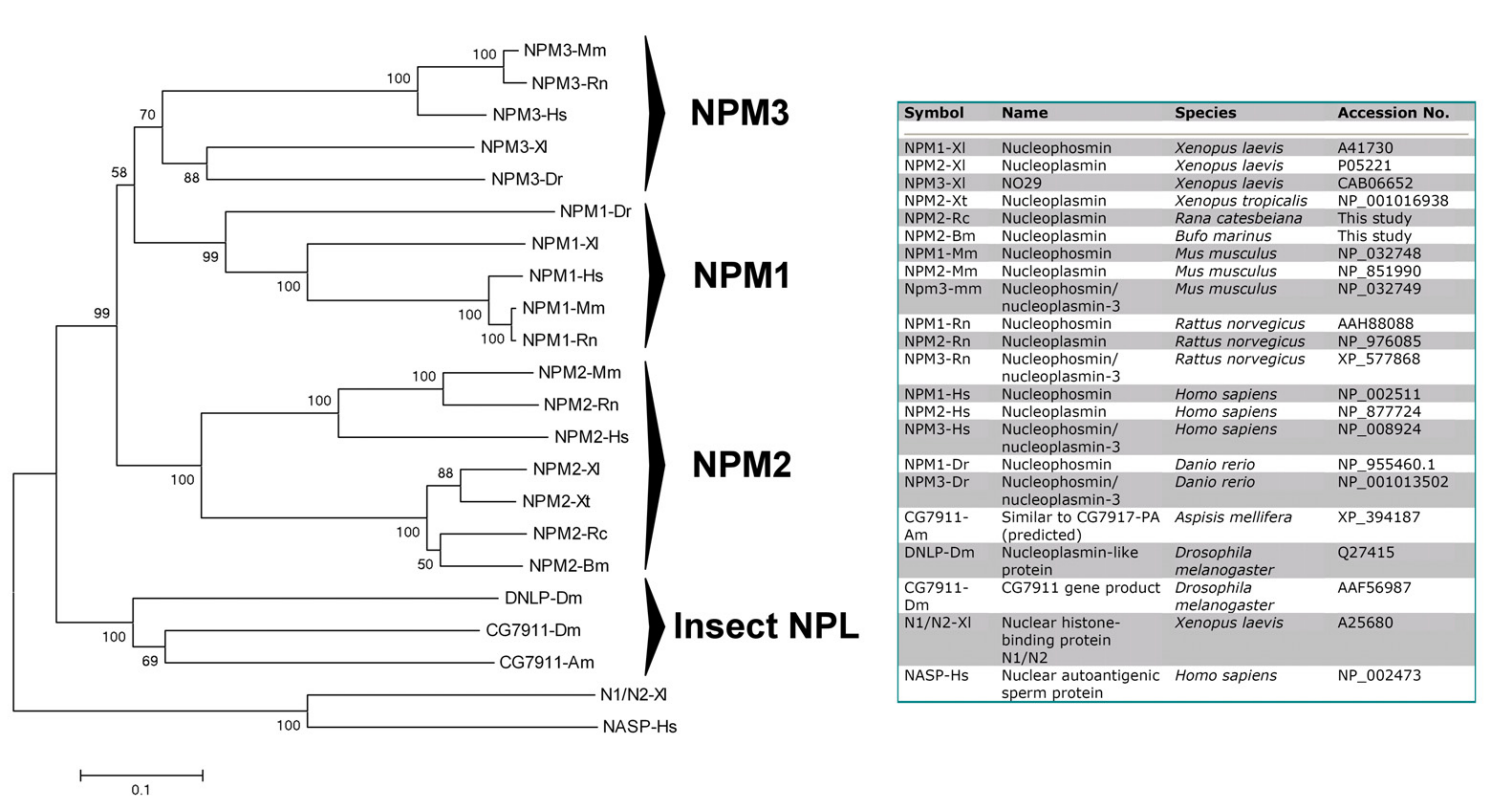
**Phylogenetic tree of nucleophosmin/nucleoplasmin family members from various metazoans**. Amino acid sequences were aligned with CLUSTAL W (Thompson et al., 1994), and the tree was produced with MEGA 3.1 (Kumar et al., 2004) using the neighbor-joining method. Two other histone-binding proteins, NASP [61] and N1/N2 [62], which are closely related to each other [61] but unrelated to the nucleophosmin/nucleoplasmin family were used to root the tree. Bootstrap significance values are shown at the corresponding internal nodes after 1000 replications.

It is possible to use the sequence information shown in Figure 5 in the context of all the nucleophosmin/nucleoplasmin metazoan sequences to determine the phylogenetic relationships of their main families: NPM1, NPM2, and NPM3 (Fig. 6). NPM1 includes a set of nucleoplasmin-like proteins, also referred to as nucleophosmin, which localize to the nucleolus of somatic cells and have been implicated numerous cellular processes including ribosome biogenesis [30], centrosome duplication [31] and nuclear chaperoning [32,33]. NPM1 variants have an important role in embryonic development and mutations of the genes encoding these proteins have been shown to be involved in acute myeloid leukemia [34]. NPM2 and NPM3 are nuclear chaperone proteins. The latter is ubiquitously expressed in many tissues [35] in contrast to NPM2 which is only found in oocytes and eggs [36]. Mammalian NPM2 has been shown to play a critical role in nucleolar and nuclear organization and plays an important role in the maintenance of the perinuclear heterochromatin which is usually observed in eggs and early embryos [36]. Although *Npm3 *antisense oligonucleotides injected into mammalian oocytes significantly prevented histone assembly and male pronuclear formation [37], the molecular components involved in the sperm chromatin remodeling after fertilization in mammals still remains largely unknown.

As shown by Figure 6 and not surprisingly, the four amphibian sequences cluster together in this analysis. They are in turn grouped with NPM2 opposed to family members in NPM1 and NPM3 or the insect nucleoplasmin-like proteins. Although amphibian nucleoplasmins group with mammalian NPM2 relative to other family members, they are in distinct clusters of the evolutionary analysis. In addition, broader evolutionary analyses (manuscript in preparation) reveal a polyphyletic origin for the NPM2 lineage, which maybe due to the differentiation between amphibians (SNBPs of H, PL, and P-types) and mammals (SNBPs of the P-type).

### *R*. catesbeiana nucleoplasmin is mainly expressed in oocytes

**Figure 7:**
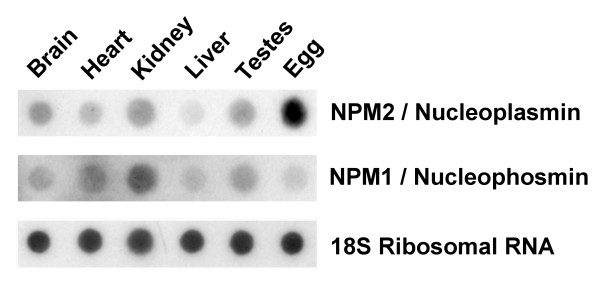
**Northern dot blot hybridizations comparing *Npm2 *and *Npm1 *mRNA levels in different *R. catesbeiana *tissues**. In the top two rows 7.5 μg of total RNA was loaded per well and the blot was probed with P^32 ^labeled *X. laevis Npm1 *or *R. catesbeiana Npm2 *cDNA amplified from PCR. In the bottom row 1.5 μg of total RNA was loaded per well and the blot was probed with a P^32 ^labeled 18S ribosome cDNA probe which was used as a loading control.

In order to put the phylogenetic implications into a functional perspective, we made RNA extracts from different tissues of *R. catesbeiana *and the levels of expression of nucleoplasmin and nucleophosmin were assessed using dot blot Northern hybridization (Fig. 7). Of the three genera of amphibians studied here, we chose *Rana *for this analysis because we reasoned that the presence of canonical histones in the mature sperm of this organism makes it unlikely that the major function of nucleoplasmin is that of remodeling of the male pronucleus chromatin.

The results obtained are similar to those obtained with other vertebrate species. As expected from its generic participation in ribosome biogenesis, nucleophosmin was widely distributed throughout the different tissues analyzed (Fig. 7) with different levels of expression which likely reflect their ribosome assembly nucleolar activity. In contrast, nucleoplasmin (Fig. 7) mRNA appeared to be primarily transcribed in the egg, a result which is consistent with the results reported for other species [9,36]. Although some background is observed in the lane of dots corresponding to the nucleoplasmin tissue distribution, this appears to follow the same pattern of distribution observed for nucleophosmin and it is likely the result of the some cross hybridization of the nucleoplasmin and nucleophosmin probes.

Thus, like mammalian nucleoplasmin, amphibian NPM2 is predominantly expressed in the oocyte nuclei were it likely accumulates and persists throughout the first stages of early development [36]. In the case of mammals, NPM2 participates in different roles from nucleolar organization to chromatin remodeling [36].

## Discussion

**Figure 8:**
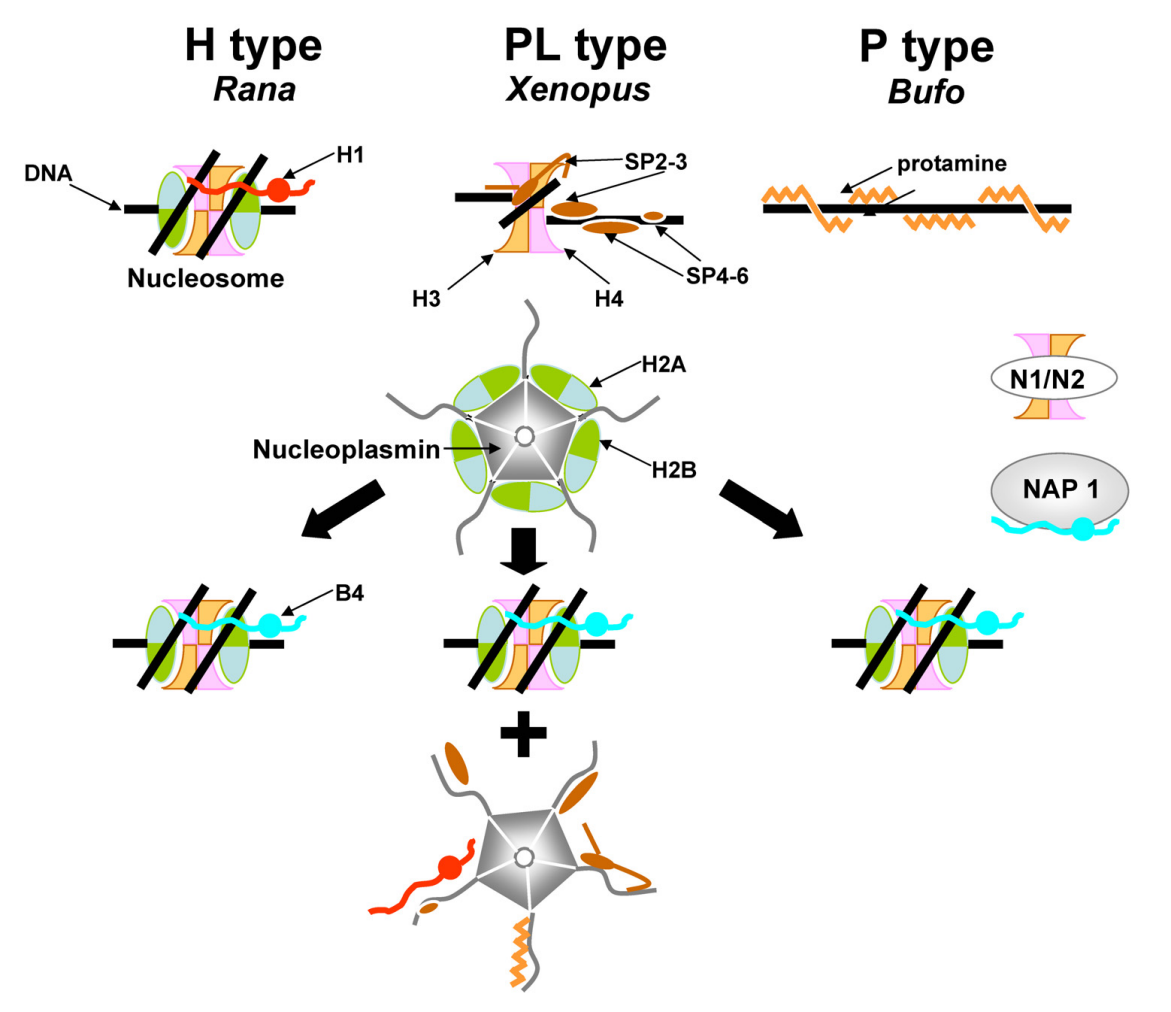
**Schematic representation of the amphibian sperm chromatin remodeling by nucleoplasmin**. The chromatin structures corresponding to each of the different SNBP types is schematically shown in the upper part of the figure. Upon fertilization of the egg, nucleoplasmin (shown here as a pentamer) stereo-specifically bound to histone H2A-H2B dimers exchanges these dimers with the SNBP components that become non-specifically (electrostatically) bound to the polyglutamic tracts of the unstructured C-terminal tails of the molecule. Note that only chromosomal proteins associated with the "linker-like" (non-helically constrained) domains of the sperm chromatin are extracted by nucleoplasmin which is highly phosphorylated at this stage of development [39]. Other nuclear chaperones are likely involved in the transition from the sperm chromatin to the male pronuclear chromatin. In this regard N1/N2 would be responsible for the assembly of H3/H4 [6] and NAP-1 for the assembly of egg/early embryo-specific histone B4 like histone H1 molecules [45].

In the introduction we put forward a question regarding the specificity of nucleoplasmin within the functional context of its participation in the remodeling of sperm chromatin with different types of SNBPs (see Fig. 8).

The results described in the previous section show that within amphibians, nucleoplasmin is a highly conserved protein (Fig. 2 and Figs. 4, 5) in contrast to the structural and compositional heterogeneity of the SNBPs (Fig. 1). This suggests that the removal of SNBPs is a highly non-specific process, a notion that is further reinforced by the observation that nucleoplasmin binds equally well to protamines [25,38], protamine-like proteins [9,38,39] and somatic linker histones [39]*in vitro*. The non-specificity of the sperm chromatin remodeling process (Fig. 8) is also supported by the observations that different egg extracts appear to be interchangeable in terms of their ability to decondense sperm chromatin[7]. In addition, the normal progression of sperm DNA decondensation in NPM2-null embryos in mice suggests that other related proteins (either NPM1 or NPM3) may even be able to compensate in this process [36]. It is possible that the polyglutamic tracts of nucleoplasmin in conjunction with phosphorylation play a critical role in out-competing the electrostatic interaction between the highly positively charged SNBPs and DNA in sperm chromatin [9].

The lack of specificity with which nucleoplasmin removes SNBPs during the formation of the male pronuclei (Fig. 8) contrasts with the specificity implicit in the binding of H2A and H2B histones during the early stages of development of the zygote. While the molecule has a conserved binding site highly specific for histones [8], which is most likely stereo-specific [40] and involves the highly structured N-terminal part of the molecule [8], the binding of SNBPs is quite non-specific and it probably involves the glutamic acid rich domains that become overexposed when the molecule becomes phosphorylated during oocyte maturation [41]. Indeed, nucleoplasmin is found associated with H2A-H2B in the egg [1], a property that originally led to the first time coining of the term molecular chaperone to describe this molecule [1,3,7]. Our results suggest that in addition to *X. laevis*, whose sperm chromatin is deficient in H2A-H2B [10], nucleoplasmin contributes the H2A-H2B dimers during the formation of the male pronuclei in other vertebrate organisms regardless of the initial SNBP composition of the male chromatin during fertilization. Other assembly factors such as N1/N2 [42-44] and NAP1 [45] provide the H3-H4 and egg-specific linker histones (such as B4 [46] in *Xenopus*) respectively. This process continues throughout the assembly of chromatin that takes place during the cell divisions preceding the mid blastula transition [7]. Nucleoplasmin phosphorylation, which remains high until this stage, may facilitate the histone exchange between nucleoplasmin and the newly replicated chromatin and does not interfere with the H1 deposition that takes place downstream of the replication fork long after the nucleosomes have been assembled [47].

Finally, we have shown that nucleoplasmin shares a close phylogenetic relationship (Fig. 6) and similar tissue distribution (Fig. 7) to mammalian NPM2 [36]. Not only this, but the genes encoding for these two proteins exhibit a highly complex similar organization which in the case of the amphibian counterpart spans over eight translated exons (Fig. 2). Thus, it is highly possible that like mammalian NPM2, amphibian nucleoplasmin has some additional chromatin remodeling functions during these early stages of development. All these observations (Fig. 2 and Fig. 6, 7) also confirm that mammalian NPM2 is the genuine mammalian equivalent of amphibian nucleoplasmin.

## Conclusion

Our results clearly show that nucleoplasmin is highly conserved between different amphibian species and its presence is independent of the SNBP type. Although amphibian nucleoplasmin clusters with mammalian NPM2 in phylogenies, broader evolutionary analyses (manuscript in preparation) reveal a polyphyletic origin for the NPM2 lineage, which maybe due to the differentiation of SNPBs between amphibians (H, PL, and P-types) and mammals (P-type). Thus, while histone storage and exchange in early development represents a critical function of nucleoplasmin, the appearance of SNBPs of the H-type early in metazoan evolution, as well as their subsequent differentiation towards PL and P-types, could have been a strong enough functional constraint to recruit SNBP removal as an acquired non-specific function. This additional role became critical in animals as the vertical evolution of SNBPs progressively led to the incorporation of more specialized PL and P proteins in sperm chromatin.

In closing, nucleoplasmin can be defined as a non-specific ATP-independent sperm chromatin remodeller with a highly specific chromatin assembly activity that plays a critical role in chromatin metabolism during the early stages of development.

## Methods

### Living organisms

*X. laevis *were reared at the University of Victoria, *B. marinus *were purchased from Wards Natural Science Ltd. (St. Catherines, Ontario) and *R. catesbeiana *from Island Bullfrogs (Nanaimo, B.C.). Investigations were conducted in accordance with the National Research Council (NRC) publication *Guide for Care and Use of Laboratory Animals *(copyright 1996, National Academy of Science) under the approval of the University of Victoria's Animal Care Committee.

### Electrophoretic analyses of SNBPs

SNBPs were extracted from testes with 0.4N HCl and precipitated with acetone as described by [48]. Proteins were separated by AU-PAGE (5% acetic acid-12% PAGE-2.5 M urea) according to [49]. AUT-PAGE (5% acetic acid-10.5% PAGE-5.25 M urea-5 mM Triton X-100) was a modified recipe from that described in [50]. The gels were prepared by mixing the following: 7 mg thiourea, 5 ml (20:1 acrylamide-bisacrylamide), 0.48 ml of glacial acetic acid, 3 g urea, 24 μl of 45 mM NH_4_OH (made fresh), 0.118 ml of 25% Triton X-100 and 1.33 ml of double distilled water. After the urea had been completely solubilized, 45 μl of 30% H_2_O_2_was added and the solution was immediately poured between the glass plates as polymerization proceeds very quickly. These gels do not need to be pre-electrophoresed and can be used immediately after polymerization. The gels were stained with Coomassie Brilliant Blue R [0.2% (w/v)] in 25%/10% (v/v) isopropanol/acetic acid and destained in 10%/10% isopropanol/acetic acid.

### Polyclonal antibodies

Rabbit polyclonal antibodies were raised against recombinant nucleoplasmin [28] which had been expressed and purified as described elsewhere [25].

### Western blot analysis with nucleoplasmin antibody

For preparation of high speed extracts enriched in nucleoplasmin, ovaries were dissected from mature females after three injections of human chorionic gonadotropin (3000 units per frog) (Sigma, Oakville, ON) given equally over 5 days. The high speed extract purification procedure followed the protocol of [51] with 1/100 v/v complete protease inhibitor (Roche Diagnostics, Laval, Qc) added to the buffers. *R. catesbeiana *nucleoplasmin was further purified by HPLC. For this, the egg high speed extract was filtered with a Nanosep MF microconcentrator (Pall Filtron Corporation, Northborough, MA) and fractionated by HPLC on a reverse phase 300-A° Vydac C18 column (25 × 3 × 0.46 cm) (Vydac, Hesperia, CA) and eluted at 1 ml/min with a 0.1% TFA-acetonitrile gradient [52].

For Western analysis, nucleoplasmin proteins or high speed extracts were separated by 10% SDS-PAGE [53], electo-transferred to polyvinylidene difluoride membrane (Bio-Rad, Mississauga, ON) and processed as described in [54]. The anti-nucleoplasmin serum was diluted 1:5000 and a secondary goat anti-rabbit horseradish peroxidase conjugate (Sigma, Oakville, ON) was diluted 1:3000.

### Mass Spectroscopy partial peptide sequences

SDS-PAGE separated *R. catesbeiana *and *B. marinus *protein bands, identified by Western Blot analysis as nucleoplasmin, were excised and the tryptic digested gel plugs analyzed on a Q-Star nanospray MS/MS analysis at the UVic Genome BC Proteomics center. Alternatively, the gel plugs were digested with Glu-C endoproteinase (*EC 3.4.21..19*) using 50 ng/uL Glu-C enzyme overnight at room temperature following the in-gel digestion and extraction protocol described by [55] and analyzed using electrospray ionization to spray the analyte into a LTQ-FTMS instrument (ThermoElectron, San Jose, CA).

### cDNA sequence obtained from RT-PCR and RACE

For RT-PCR total RNA was extracted from oocytes using Trizol reagent (GibcoBRL, Burlington, ON) and cDNA was synthesized using Superscript II RNase H^- ^reverse transcriptase (Invitrogen, Burlington, ON). Degenerate primers for PCR were created based on the determined amino acid sequences for *B. marinus *and *R. catesbeiana *and the *X. laevis *nucleoplasmin cDNA sequence. Polyadenylated mRNA was purified using the MicroPoly(A) Purist small scale mRNA purification kit (Ambion, Austin, TX) and used for 3'and 5' rapid amplification of cDNA ends (RACE) using the FirstChoice RLM-RACE Kit (Ambion, Austin TX) following the manufacturers directions. PCR was performed using a PCRsprint thermal cycler (Hybaid, Teddington, UK) with cDNA as the template. The following primers were designed for RACE from the internal sequence:

*Rana *3'RACE inner 5'-GCAAAGGATGAGTTCCACATAGTA-3'

*Rana *3'RACE outer 5'-CATCAACTGGCGTTAAGGAC-3'

*Rana *5'RACE inner 5'-ACTGCTTGAGGGACTATTTCTACTA-3'

*Rana *5'RACE outer 5'-CGGTTTTGCGTCACTCCCTTC-3'

*Bufo *3'RACE inner 5'-GATGAAGACAAGAGCGAGCA-3'

*Bufo *3'RACE outer 5'-AGCTGGCCCTGAGAACTGTA-3'

*Bufo *5'RACE inner 5'-CATCGCTCCCTTCTACCTGA-3'

*Bufo *5'RACE outer 5'-CTGGCTATAGGAACAGGTTG-5'

For DNA sequencing, agarose gel purified PCR products were cloned into pCR 2.1-TOPO vectors (Invitrogen, Burlington, ON) following the instructions of the manufacturer and transformed into TOP10 competent cells (Invitrogen, Burlington, ON). Sequencing was done by the DNA Sequencing Facility, Centre for Biomedical Research at the University of Victoria. In addition to the cDNA sequences of *R. catesbeiana *and *B. marinus*, the partial genomic DNA sequence of the *X. laevis *gene was determine to confirm that *X. laevis *had a similar intron/exon structure to *X. tropicalis*. PCR and sequencing was done as described for the cDNA using *X. laevis *genomic DNA as a template and the following PCR primers:

*Xenopus *forward inner 5'-GTGAGCATCAGTTGGCGTTGC-3'

*Xenopus *forward outer 5'-CTGGGGACAAGGCAAAGGA-3'

*Xenopus *reverse inner 5'-ACCGGAAAGTAASTGGAGGAG-3'

*Xenopus *reverse outer 5'-GAGGTTCACTTCTTAGCAGCCG-3'

### Phylogeny of nucleophosmin/nucleoplasmin family members from various metazoans

Amino acid sequences were aligned using the program CLUSTAL W [56] and the phylogenetic tree was constructed using the neighbor-joining method [57]. Amino acid sequence distances were estimated as the uncorrected differences (*p*-distance) resulting in smaller variance. The reliability of the topology was tested by the bootstrap method (1000 replications) and values are shown in the corresponding internal nodes of the topology. Two other histone-binding chaperone proteins (NASP and N1/N2), which are closely related to each other but unrelated to the nucleophosmin/nucleoplasmin family were used to root the tree. All the molecular evolutionary analyses were conducted using the computer program MEGA version 3.1 [58].

### Northern dot blot hybridizations comparing *Npm2 *and *Npm1 *mRNA levels in different *R. catesbeiana *tissues

Total RNA was extracted from oocytes using Trizol reagent (GibcoBRL, Burlington, ON) and the purity and concentration were determined by UV absorbance and agarose gel electrophoresis of denatured samples. Total RNA samples (7.5 μg for *Npm1 *and *Npm2 *or 1.5 μg for 18S ribosomal) were dissolved in 10 mM NaOH, 1 mM EDTA and transferred to Zeta-Probe GT Blotting membrane (BioRad, Mississauga, ON) using a Bio-Dot Microfiltration Apparatus (BioRad, Mississauga, ON) following the manufactures directions, then crosslinked to the membrane with UV (120 000 μJ). The cDNA probes used were produced by PCR using the following primers:

18S forward 5'-TGCATGGCCGTTCTTAGTTGGTGG-3',

18S reverse 5' CACCTACGGAAACCTTGTTACGAC-3',

NPM2 forward 5'-TCCAGAATCTCCTCCAAAACC-3',

NPM2 reverse 5'-AGGGCTTCCTTCCTCTTCCT-3'

NPM1 forward 5'-CTCAAAAGTYRAATCACAATGG-3'

NPM1 reverse 5'-TGTCTCCATTKCCARAGATCTT-3'

NPM 1 and 2 primers were designed based on nucleotide regions unique to each sequence. The 18SCOMF and 18SCOMR primers were from [59]. The 18S and *Npm2 *probes were from *R. catesbeiana *and the *Npm1 *probe was from *X. laevis*. PCR products run on agarose gels gave single bands of the expected sizes which were excised, purified using the QIAquick Gel extraction kit (Qiagen, Mississauga, ON) and sequenced as described above to confirm their identity. The purified PCR products were labeled with the Random Primer DNA Labeling System (Invitrogen, Burlington, ON) following the Standard Labeling protocol to produce probes. Hybridization of ~ 10^6^cpm ^32^P-labeled cDNA probes in PerfectHyb^tm ^Plus buffer (Sigma, Oakville, ON) were incubated overnight with the blots. Membranes were then washed to high stringency, exposed and analyzed.

## Abbreviations

AUT, Acetic acid-Urea-Triton X-100

DAPI, 4,6-diamidino-2-phenylindole;

DTT, dithiothreitol;

EDTA, ethylenedinitrilo-tetraacetic acid;

HAP, hydroxyapatite;

HEPES, N-2-Hydroxyethylpiperazine-N'-2-ethanesulfonic acid;

HP1α, heterochromatin protein 1 alpha;

MBT, mid-blastula transition;

NASP, nuclear autoantigenic sperm protein

NCP, nucleosome core particle;

NPM, nucleoplasmin (like) protein

PAGE, polyacrylamide gel electrophoresis;

PL, protamine-like;

RACE, rapid amplification of cDNA ends;

SDS, sodium dodecyl sulphate;

SNBP, sperm nuclear basic protein.

## Authors' contributions

LJF and JA conceived, designed and carried out the experiments and prepared the manuscript. JMEL participated in the evolutionary analysis and assisted with the manuscript. EDF and DFH participated in the mass spectroscopy partial peptide sequence analysis and contributed to the discussion section of the manuscript. All authors read and approved the final manuscript.
